# Using Single-Particle Fluorescence Microscopy to Quantify Substrate Binding of Peptidoglycan-Modification Enzymes

**DOI:** 10.21769/BioProtoc.5696

**Published:** 2026-05-20

**Authors:** Carlos A. Ramírez Carbó, Beiyan Nan

**Affiliations:** 1 Department of Biology, Texas A&M University, College Station, TX, USA; 2 The Genetics and Genomics Interdisciplinary Program, Texas A&M University, College Station, TX, USA

**Keywords:** Peptidoglycan, Peptidoglycan synthases, Peptidoglycan hydrolases, Single particles, Fluorescence microscopy, Single-particle tracking, sptPALM

## Abstract

Peptidoglycan (PG), a network of glycan strands crosslinked by short peptides, is an essential and bacterial-specific structure that determines cell shape and protects cells from lysis. Understanding how bacteria assemble, maintain, and modify their PG not only addresses fundamental questions in cell biology but also provides a basis for developing strategies to treat bacterial infections. Although several in vitro methods, such as zymography, Remazol Brilliant Blue (RBB) assay, and LC-MS analyses, are available to quantify the activities of PG-modification enzymes, these approaches are not readily applicable in vivo. Here, we describe a single-particle tracking photo-activated localization microscopy (sptPALM)-based method to quantify the binding of enzymes to PG in vivo, which serves as a proxy for their enzymatic activities. Because the PG meshwork is relatively immobile, fluorescently tagged enzymes that transiently or stably bind it exhibit reduced mobility, reflected by lower diffusion coefficients. This approach provides sensitive, quantitative, and real-time insights into enzyme behavior in vivo under diverse physiological conditions or genetic backgrounds. The protocol is particularly valuable for investigating PG-modification enzymes that are essential or functionally redundant, which are often difficult to analyze using traditional genetic methods.

Key features

• Builds upon the method developed by Fu et al. [1] for single-particle tracking with high spatial and temporal resolution.

• Streamlined imaging and data processing workflow.

• Automatic analysis on large datasets with minimum human bias.

• Reveals the regulatory relationship between PG-modification enzymes, bypassing complex genetic experiments.

## Graphical overview


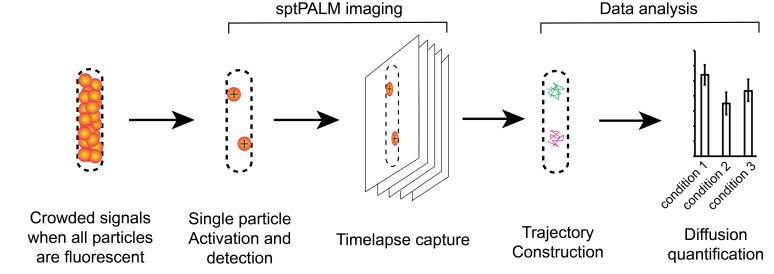


**Overview of the imaging and data analysis procedures**

## Background

The peptidoglycan (PG) cell wall is a crosslinked polymer network that surrounds most bacterial cells, defines their morphology, and protects them against osmotic lysis. This polymer is made of muropeptide units, which consist of two amino sugars, N-acetylglucosamine (NAG) and N-acetylmuramic acid (NAM), with a peptide side chain that contains unique D-amino acids attached to the latter. The sugar subunits are polymerized into glycan strands by glycosyltransferases (GTases) via β-1,4 glycosidic bonds, and adjacent glycan strands are crosslinked by transpeptidases (TPases) through their peptide moieties [5]. While class A penicillin-binding proteins (aPBPs) contain both GTase and TPase domains, SEDS (shape, elongation, division, and sporulation) proteins are monofunctional GTases that work in concert with monofunctional TPases called class B PBPs (bPBPs) [6]. Additionally, many bacteria utilize LD-transpeptidases (LDTs), which are structurally distinct from PBP transpeptidase domains and facilitate different crosslinking reactions [7].

Because the entire PG layer of each bacterial cell is a covalently closed single molecule, PG expansion, e.g., the incorporation of new glycan strands, requires hydrolases, also known as autolysins, to generate openings in the existing PG network. Endopeptidases (EPs) are particularly important as “space-making” enzymes that cleave peptide crosslinks and thus allow such insertion [8–15]. Bacteria constantly remodel their PG using large repertoires of autolysins. Each bond in the PG matrix has a specific type of enzyme with the ability to break it: besides EPs, lytic transglycosylases (LTGs) break glycan strands, while amidases and carboxypeptidases (CPs) remove amino acids from different termini of peptides [10,15–19].

Quantitative analysis of the activities of PG-modification enzymes has been a major challenge in the field. PG labeling using fluorescence-conjugated D-amino acids [20,21], carbohydrates [22,23], and antibiotics [24,25] allows in situ probing of newly synthesized PG in live cells. In addition, high-performance liquid chromatography (HPLC), ultrahigh-performance liquid chromatography (UPLC), and mass spectrometry on purified, muramidase-digested PG sacculi produce high-resolution muropeptide profiles that provide quantitative architectural information, including muropeptide composition, glycan chain length, and the crosslinks between them [3,26,27]. However, rather than monitoring individual enzymatic activities, both methods reflect the combined activities of many PG-modification enzymes.

Many in vitro assays have been developed to study individual enzymes. For instance, purified lipid II, a PG precursor, after being labeled with fluorescent dyes or radioactive isotopes, can be used to quantify the activities of purified GTases [6,28]. Zymogram and Remazol Brilliant Blue (RBB) assays are widely used on autolysins. Zymogram runs denatured autolysins in SDS-polyacrylamide gels supplemented with PG as a substrate. After electrophoresis, the gel is transferred to a renaturation solution. The refolded autolysins then degrade PG, and their activities can be reflected by the clearing zones on gels [29]. In contrast, the RBB assay quantifies the release of RBB, a dye that covalently attaches to PG on its sugar moieties. Autolysins can degrade the insoluble, RBB-modified PG and thus release the dye into solution, whose absorbance can be readily measured using a spectrophotometer [2,3,30,31]. However, as most PG-modification enzymes reside in the membrane and periplasm, these techniques face significant hurdles in protein expression, solubilization, and refolding. Moreover, the techniques for lipid II purification and labeling are not available for most microbiology laboratories.

Single-particle microscopy is a powerful imaging technique in bacterial cell biology. Due to the diffraction of light, each fluorophore, no matter how small, generates a signal that covers an area of 300–500 nm in diameter [32]. Because conventional fluorescence microscopy detects all fluorophores in the sample, hundreds to thousands of such signals inside a small bacterial cell (typically ~1 μm wide) create a blurry picture, where the dynamics of single protein particles cannot be resolved [33]. Using single-particle tracking photo-activated localization microscopy (sptPALM), only a few fluorophores are randomly switched on in each cell, enabling accurate determination of their locations. Capturing time-lapse images, we can track the movement of individual particles with high spatial and temporal resolution (~160 nm and up to 10-ms, e.g., 100 frames/s) and quantify their distribution in subcellular regions [1–4,34–36]. Here, we refer to isolated, single fluorescent spots under sptPALM as “single particles” rather than “single molecules” because they could represent protein complexes [37,38].

Here, we describe a sptPALM-based method to quantify the binding of enzymes to PG in vivo, which serves as a proxy for their enzymatic activities. Biochemical reactions on the PG scaffold are unique due to the stark size difference between the enzymes and their substrates. While enzyme particles are in nanometer scales, their substrates, the PG sacculi, span several micrometers. Under the microscope, PG remains stationary, but PG-related enzymes are free to move. When diffusive enzymes bind to PG, their mobility decreases. Consequently, increased enzyme binding to PG under specific growth or genetic conditions leads to a decrease in its diffusion coefficient. Using this method, the diffusion of many PG-modification enzyme particles, including both synthases and autolysins, has been documented [3,4,39,40]. In these studies, the diffusion coefficient (D) was used to quantify particle mobility, where high D values indicate high particle mobility and thus reflect low PG binding. However, the negative correlation between D and PG binding is not necessarily linear.

Much of our knowledge on PG assembly and modification is based on mutant phenotypes. However, many SEDS and their partner bPBPs are essential and thus undeletable. In addition, aPBPs and PG hydrolases are notoriously redundant in most bacteria, where strains lacking single or several of these enzymes rarely display significant phenotypes (*B. subtilis* remains viable when 41 of the 42 PG hydrolases are absent) [10,17,41–44]. Phenotypes, if they exist, are unreliable for assigning functions to PG-related enzymes, because even when two enzymes have distinct functions, their null mutants could produce similar phenotypes [4,16]. This method captures the diffusion of hundreds to thousands of protein particles, enabling us to elucidate how PG-modification enzymes regulate each other in live cells, even in the absence of pronounced “macro-phenotypes” in morphology, growth, or division. For this reason, this protocol is especially useful for studying the PG-modification enzymes that are either redundant (such as some aPBPs and most autolysins) or essential (such as the Rod components).

## Materials and reagents

**Bacterial strains**

As single-particle imaging has been successfully applied to many bacteria, this protocol is adaptable to all bacteria that express photo-activatable fluorophore-labeled proteins. Here, we use the Gram-negative bacterium *Myxococcus xanthus* to demonstrate the protocol. *M. xanthus* is a model organism for studying many functions in bacteria, including PG dynamics [45,46]. The *M. xanthus* BN312 strain expresses photo-activatable mCherry (PAmCherry)-labeled DacB using the native *dacB* locus and promoter [4]. BN312 was constructed in the wild-type *Myxococcus xanthus* DZ2 [46–48] background. Methods describing how to generate stable strains of *M. xanthus* expressing PAmCherry have been published elsewhere [1–4,49]. Both DZ2 and BN312 are available upon request. This protocol uses PAmCherry as an example for sptPALM. Numerous other photo-activatable proteins and synthetic dyes are available. Many equipment and chemical suppliers provide extensive guides on their biological and optical properties, such as https://www.leica-microsystems.com/science-lab/life-science/photoactivatable-photoconvertible-and-photoswitchable-fluorescent-proteins/ and https://app.fluorofinder.com/dyes.

**Reagents**

1. Agarose RA^TM^ (VWR Life Science, CAS: 9012-36-6)

2. MOPS (Thermo Fisher Scientific, CAS: 1132-61-2)

3. KOH (Thermo Fisher Scientific, CAS: 1310-58-3)

4. Bacto^TM^ Casitone (Thermo Fisher Scientific, CAS: 9000-71-9)

5. BD yeast extract (Sigma-Aldrich, CAS: 8013-01-2)

6. MgSO_4_ (Sigma-Aldrich, CAS: 7487-88-09)

7. Antibiotics

*Note: Many antibiotics target peptidoglycan enzymes, such as moenomycin, which inhibits the GTase activities of the aPBPs, and mecillinam, which blocks the TPase activity of the Rod system. Choose antibiotics based on the targets and determine their working concentrations based on phenotypes and minimum inhibitory concentrations (MICs). In this protocol, we use moenomycin at 4 μg/mL, which does not stop* M. xanthus *growth but turns rod-shaped cells into spheres [4].*

**Solutions**

1. 0.8% agarose gel (see Recipes)

2. 1 M MOPS pH 7.6 (see Recipes)

3. 0.8 M MgSO_4_ (see Recipes)

4. CYE medium (See Recipes)

**Recipes**

**1. 0.8% agarose gel (w/v)**

**Table:** 

Reagent	Final concentration	Quantity or volume
Agarose	0.8% (w/v)	8.0 g
1 M MOPS pH 7.6	10 mM	10 mL
ddH_2_O	n/a	980 mL
Total	n/a	1 L

*Note: Agarose concentration can be modified according to the specific requirements of each organism. However, a concentration lower than 0.4% makes the agarose pad unstable. The buffer system, its concentration, and pH can be modified according to the specific requirements of each organism. Autoclave to sterilize, aliquot to 15 mL centrifuge tubes, tighten the tube caps, and store at room temperature. In our experience, such premade agarose gel remains stable for 3 months.*

**2. 1 M MOPS pH 7.6**

**Table:** 

Reagent	Final concentration	Quantity or volume
MOPS powder	1 M	209.27 g
ddH_2_O	n/a	980 mL
Total	n/a	1 L

*Note: First, dissolve 209.27 g of MOPS powder into 600–800 mL of ddH_2_O. The initial pH is about 5. Adjust pH with KOH. Begin with pellets or a 10 N KOH stock solution and then slow down when the pH is approaching 7.6. After pH is adjusted, adjust the volume to 1 L by adding ddH_2_O. A cylinder is required. Filtrate the final solution to sterilize. Wrap the bottle with foil and store it at 4 °C. MOPS solution is sensitive to light. It will turn yellow when exposed to light.*

**3. 0.8 M MgSO_4_**

**Table:** 

Reagent	Final concentration	Quantity or volume
MgSO_4_ powder	0.8 M	96.3 g
ddH_2_O	n/a	980 mL
Total	n/a	1 L

*Note: After the MgSO_4_ powder is dissolved in ddH_2_O, adjust the volume to 1L. Autoclave immediately after preparation.*

**4. CYE medium**

**Table:** 

Reagent	Final concentration	Quantity or volume
BD Casitone	1% (w/v)	10 g
BD yeast extract	0.5% (w/v)	5 g
1 M MOPS pH 7.6	10 mM	10 mL
0.8 M MgSO_4_	8 mM	10 mL
ddH_2_O	n/a	390 mL
Total	n/a	1 L

*Note: Autoclave immediately after preparation. CYE medium was used in this protocol to grow* M. xanthus. *For other organisms, choose appropriate media according to their growth requirements.*

**Laboratory supplies**

1. 15 mL centrifuge tubes, Nunc^TM^ conical sterile polypropylene centrifuge tubes (Thermo Fisher Scientific, catalog number: 339650)

2. Fisherbrand^TM^ Selectfrost^TM^ microscope slides (Fisher Scientific, catalog number: 1255001)

3. VMR micro cover glasses, square, no. 1 1/2 (VWR, catalog number: 48366-249)

4. Pipettes (20-μL, 200-μL, and 1,000-μL) and tips

## Equipment

1. Eppendorf BioPhotometer model #6131 (or comparable brands and models)

2. VWR Incubator Model #1920 (or comparable brands and models)

3. Inverted Nikon Eclipse-Ti^TM^ microscope with a 100× 1.49 NA TIRF objective

*Note: PAmCherry is activated using a 405-nm laser (0.3 kW/cm^2^), and excited and imaged using a 561-nm laser (0.2 kW/cm^2^).*

4. Hamamatsu ImagEM X2^TM^ EM-CCD C9100-23B or Andor iXon Ultra^TM^ 897 EMCCD camera

*Note: These experimental procedures, though demonstrated on the above equipment, are adaptable to a wide range of microscopy and camera configurations. A total internal reflection fluorescence (TIRF) objective is required for reducing background noise in single-particle microscopy. Under the TIRF setup, only a section of ~200 nm of the cells close to the coverslip is illuminated, which includes the space where PG-modification enzymes reside. When paired with a 100× 1.49 NA TIRF objective, both cameras provide an effective pixel size of 160 nm. Pixel sizes must be entered into the MATLAB data analysis script in accordance with the hardware utilized.*

## Software and datasets

1. We developed a MATLAB-based script for single-particle data analysis (https://github.com/NanLabMyxo/Rod_shape_paper) under the “/Matlab 1 population” folder [1,4].

2. MATLAB R2018b (The MathWorks, Inc. https://www.mathworks.com/help/install/ug/install-products-with-internet-connection.html)

3. Optimization Toolbox (https://www.mathworks.com/products/optimization.html)

4. NIS-Elements AR 5.30.02 64-bit (https://www.microscope.healthcare.nikon.com/products/software/nis-elements/software-resources)

*Note: Single-particle imaging, although demonstrated using NIS-Elements^TM^, is adaptable to a wide range of software.*

## Procedure

**A. Sample preparation**

1. Cell growth: Grow the cells in liquid media to an approximate concentration of OD_600_ 1 with proper antibiotics and/or supplements.

*Note: In this protocol, 25 mL of CYE liquid media was used to grow BN312, an* M. xanthus *strain, in a 125-mL flask at 32 °C. For other organisms, choose appropriate media and volumes according to their growth requirements. For example*, Escherichia coli *grows well in 2–5 mL of LB medium in test tubes at 37 °C.*

**Figure 1.**
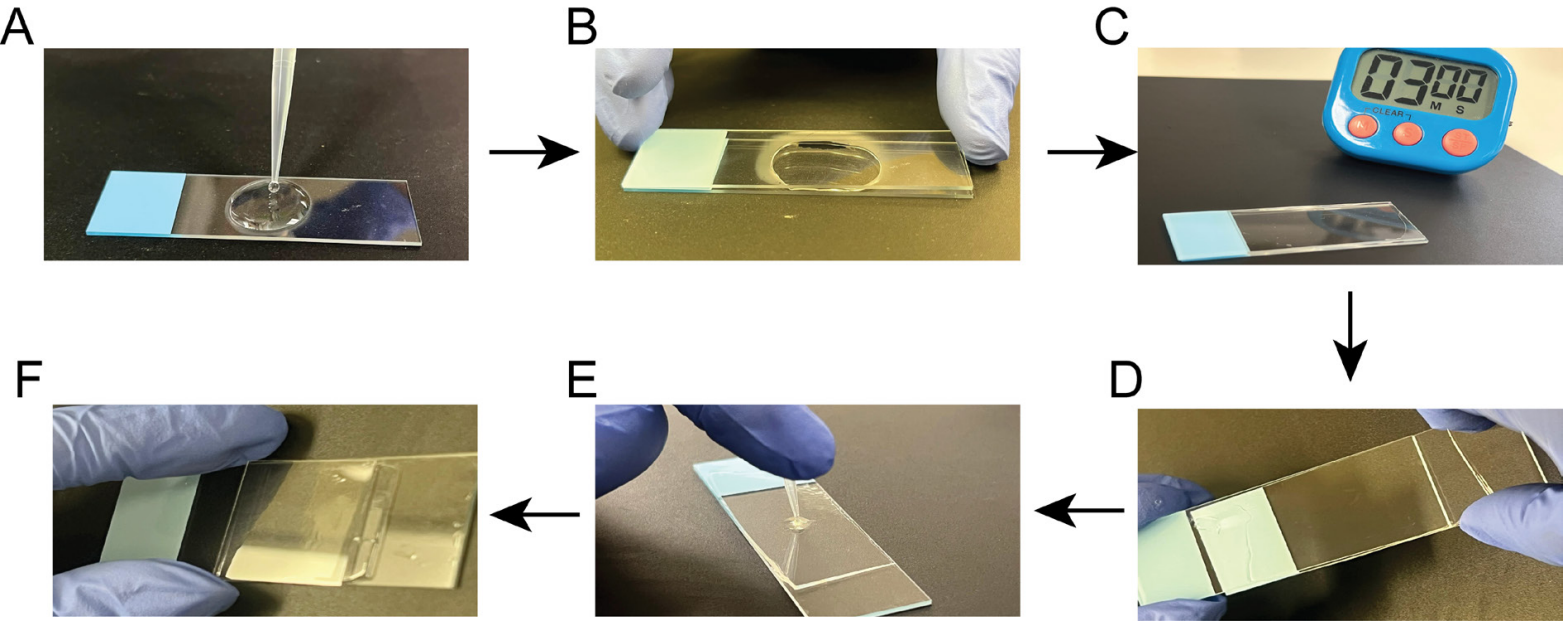
Steps for making an agarose pad. (A) Pipette 0.6 mL of melted agarose onto a microscope slide. (B) Gently place a second slide on top to spread the agarose evenly. (C) Allow the agarose to solidify for 2–3 min. (D) Carefully separate the slides, leaving a ~1 mm thick agarose pad on one slide. (E) Apply 5 µL of liquid cell culture onto the pad. (F) Gently place a coverslip over the agarose pad to complete the preparation.

2. Agarose pad preparation:

a. Use a microwave to melt the 0.8% agarose gel.

b. Drop 0.6 mL of melted agarose onto a microscope slide (Figure 1A) and gently place another microscope slide on top of the drop (Figure 1B).

*Note: When necessary, nutrients and inhibitors, such as antibiotics, can be added to the agarose pad.*

c. Wait 2–3 min for the agarose to solidify (Figure 1C).

d. Gently separate the two microscope slides. An agarose pad of ~1 mm thickness will remain on one of the slides (Figure 1D).

3. Add 5 μL of liquid cell culture on the agarose pad and wait 3–5 min for it to air dry (Figure 1E).

4. Gently place a coverslip on top of the agarose pad (Figure 1F).

*Note: Avoid bubble formation by holding the coverslip as close as possible to the agarose pad before placing it onto the agarose pad. Use caution and avoid sudden movements.*

**B. Single-particle imaging**

1. Place the slide in the microscope and focus on the sample.

2. Launch NIS-Elements AR 5.30.02 (64-bit).

*Note: This software is available for Nikon microscopes. Microscopes from other manufacturers use different software with comparable settings.*

3. Select the differential interference contrast (DIC) channel to visualize cells.

*Note: In this protocol, we take brightfield images using the DIC channel. However, our analysis script can also recognize phase contrast images.*

**Figure 2.**
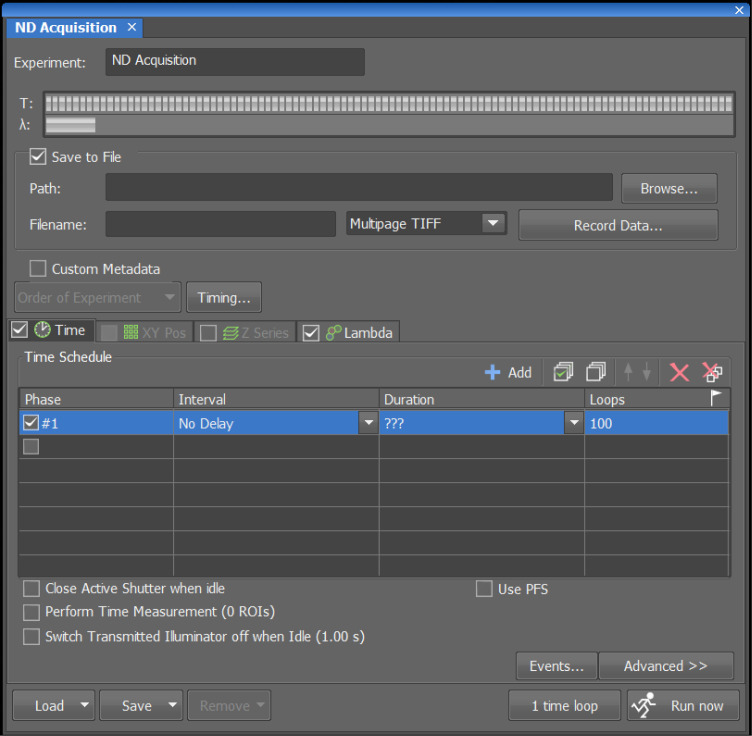
Software settings for taking 100 images from each field

4. Open ND Acquisition:

a. Under *Time*, set *Interval* to “no delay” and *Loops* to 100 (take 100 images from each field) (Figure 2).

*Note:* Interval *refers to the time gap between images.* No delay *indicates that images will be acquired continuously, with no delay between images. This value can be changed. For instance, if the user plans to acquire one image every second with a 100-ms exposure for each frame, the* Interval *will be 900 ms.* Duration *will be calculated automatically after setting the interval and loops.*

**Figure 3.**
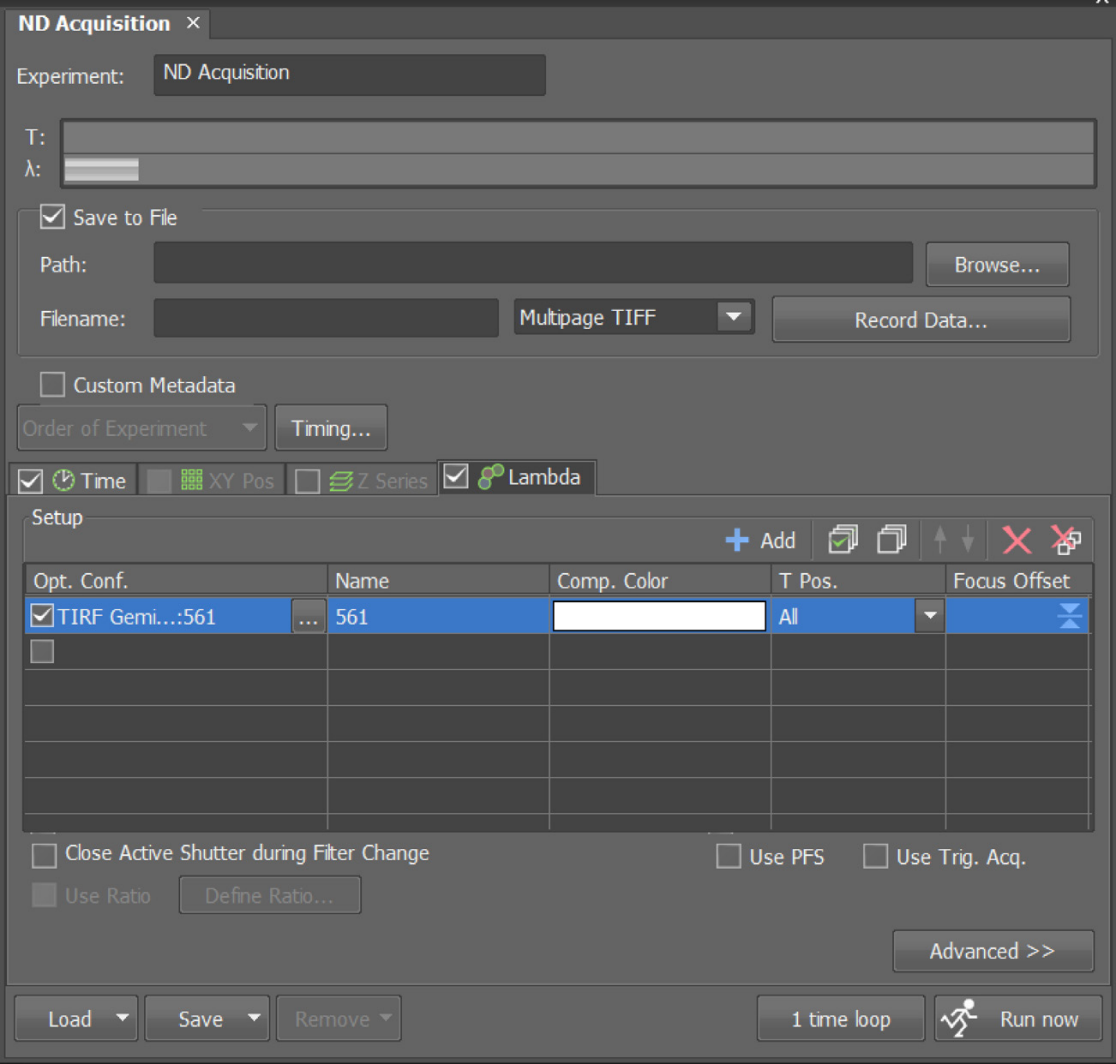
Setting the excitation wavelength for PAmCherry

b. Under *Lambda*, select the *561-nm laser* in *Opt. Conf.* to enable automatic activation during imaging (Figure 3).

**Figure 4.**
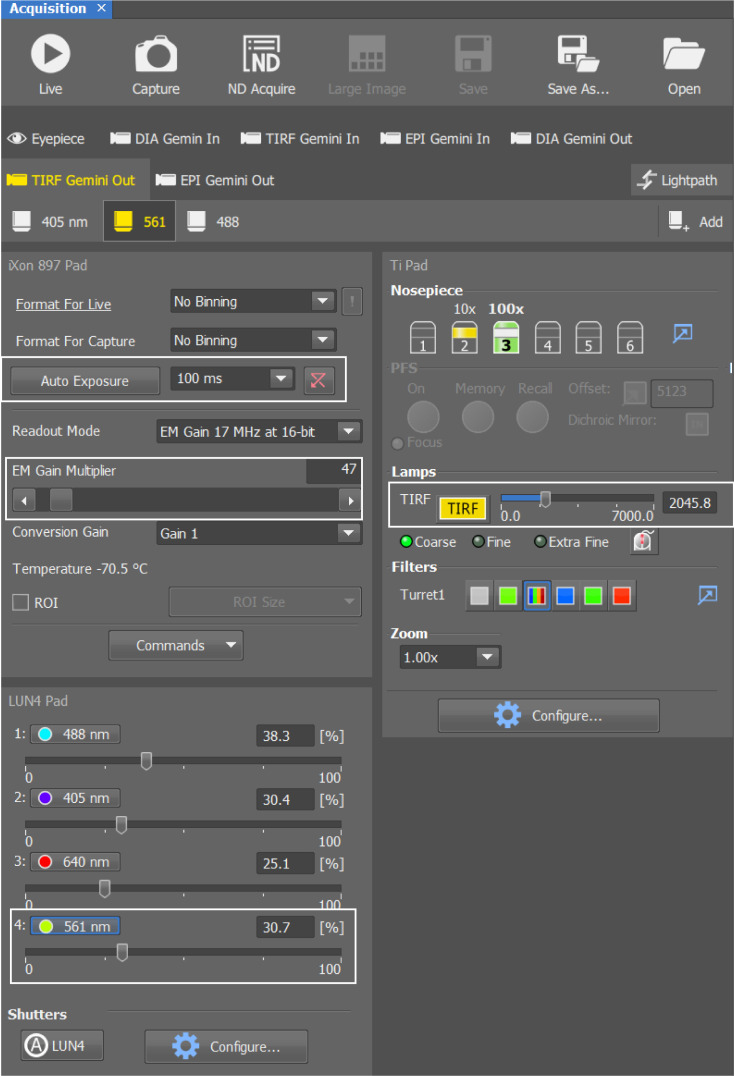
Software settings for imaging activated PAmCherry, including exposure time, laser intensity, camera gain, and TIRF angle (white boxes)

5. Go to the fluorescent channel, select the 561 nm laser, adjust laser intensity, and set the exposure time to 100 ms (Figure 4). With a 100-ms exposure time and no delay between images, the imaging frequency is 10 Hz, and the temporal resolution is 100 ms.

*Note: A 561-nm laser is used to excite the activated PAmCherry particles. Different fluorophores require different excitation wavelengths. Please refer to their optical properties, which can be easily found online, such as* *https://www.leica-microsystems.com/science-lab/life-science/photoactivatable-photoconvertible-and-photoswitchable-fluorescent-proteins/* . *Using our settings, we were able to track the free diffusion of single PAmCherry particles using 10-ms exposure and thus image at 100 frames/s [1].*

6. In the DIC channel, identify a field containing at least 10 well-separated cells to facilitate MATLAB-based analysis.

7. Take a brightfield image of cells.

*Note: The brightfield image will be used by the data analysis script for cell identification.*

8. Switch to the fluorescence channel and select the 405-nm laser to activate PAmCherry molecules. Limit exposure to 2–3 s to minimize photobleaching. Activation time and laser power may vary for different proteins and organisms.

9. After activation, switch to the 561-nm laser and initiate acquisition using the predefined ND Acquisition settings by clicking *Run Now*. This records fluorescence emission at 10 Hz, allowing visualization of individual molecule dynamics.

*Note: When necessary, adjust TIRF angle for best contrast. PAmCherry-labeled protein particles are randomly activated to their fluorescent state. The number of proteins that are activated can be adjusted by tuning the power and activation time of the 405-nm laser. Also, tune the exposure time and camera gain using the 561-nm laser to achieve the best signal-to-noise ratio. To achieve optimal results, ensure an average activation density of 1–2 particles per cell. Different photo-activatable fluorophores require different excitation wavelengths. Adjust the settings in step B5 (including laser intensity and camera gain) and the activation time and intensity of the 405-nm laser in step B8 until single particles are clearly visible. Use a new field for each adjustment cycle.*

10. Save the time-lapse image stack as a single .tiff file. Video 1 displays a typical time-lapse stack of DacB-PAmCherry particles in BN312 cells.

**Video 1.**
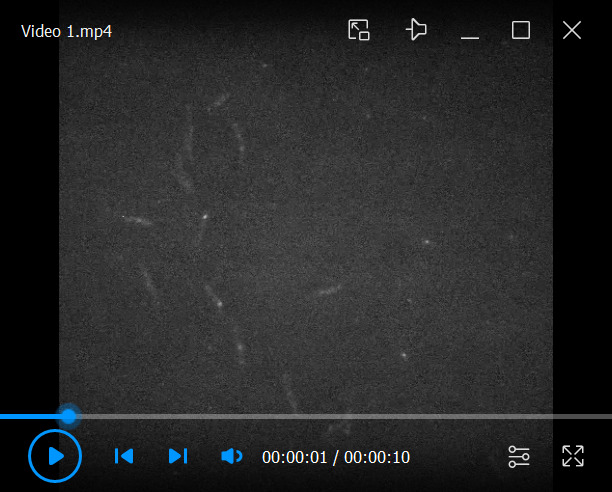
Dynamics of DacB-PAmCherry particles in BN312 cells

11. Move to a new field and repeat this imaging sequence until you have imaged a few hundred individual cells.

*Note: To reduce the number of imaging sequences, try to image the fields that contain many relatively isolated cells. To obtain confident information, we recommend collecting data from at least 100 cells from three biological replicates, which will generate mobility information of hundreds to thousands of particles.*

**C. Calculating diffusion coefficient**

**Figure 5.**
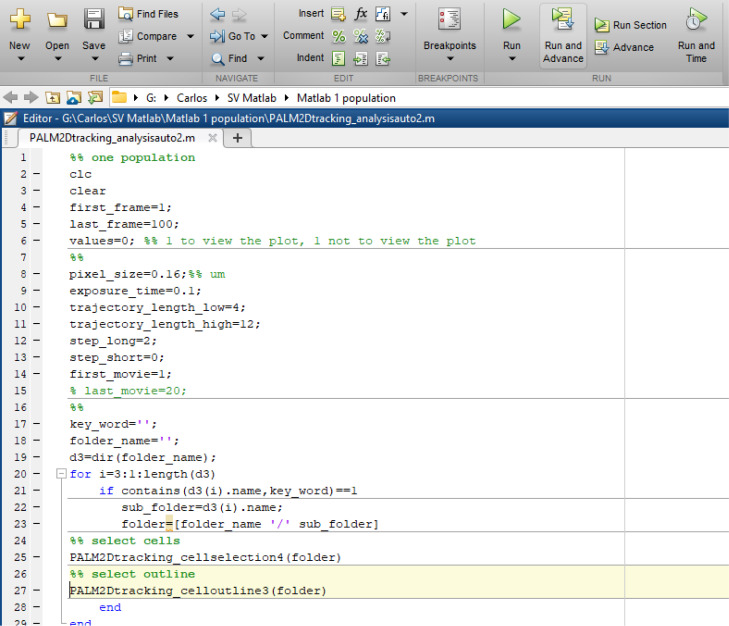
Interface of the MATLAB script

1. Start MATLAB and load the “PALM2Dtracking_analysisauto2.m” script (Figure 5).

2. In lines 4–5, type 1 after first_frame= and 100 after last_frame=.

*Note: These settings define the total number of images in each time-lapse data set.*

3. In line #8, after pixel_size=, enter the pixel size in micrometers.

*Note: The pixel size is a value specific to the combination of the camera and objective. This value can be measured using stage microscope slides (such as the AmScope MR095 Stage Micrometer Calibration Slide, Model WBM1787816 or obtained from the manufacturer).*

4. In line #9, after exposure_time=, enter exposure time in seconds.

*Note: Exposure time is reciprocal to the imaging frame rate. For example, if the imaging frame rate is 10 frame/s, then enter 0.1 here.*

5. Lines 10 and 12 set the thresholds for data analysis. Enter 4 after trajectory_length_low= in line 10 and 12 after trajectory_length_high= in line 11.

*Note: Based on our experience, we recommend these settings because most fluorescent particles remain in focus for 4–12 frames.*

6. In line 17, after key_word=, enter one part of the directory name (letters, numbers, or both) that contains the images.

*Note: The program will use this entry to identify and load the correct folder. Make sure to use single quotation marks (') around the key_word. For example, if the directory name is “20260203_DacB_PAmCherry,” then the key_word could be ‘2026,’ ‘0203,’ ‘DacB,’ ‘PAmCherry,’ etc.*

**Figure 6.**
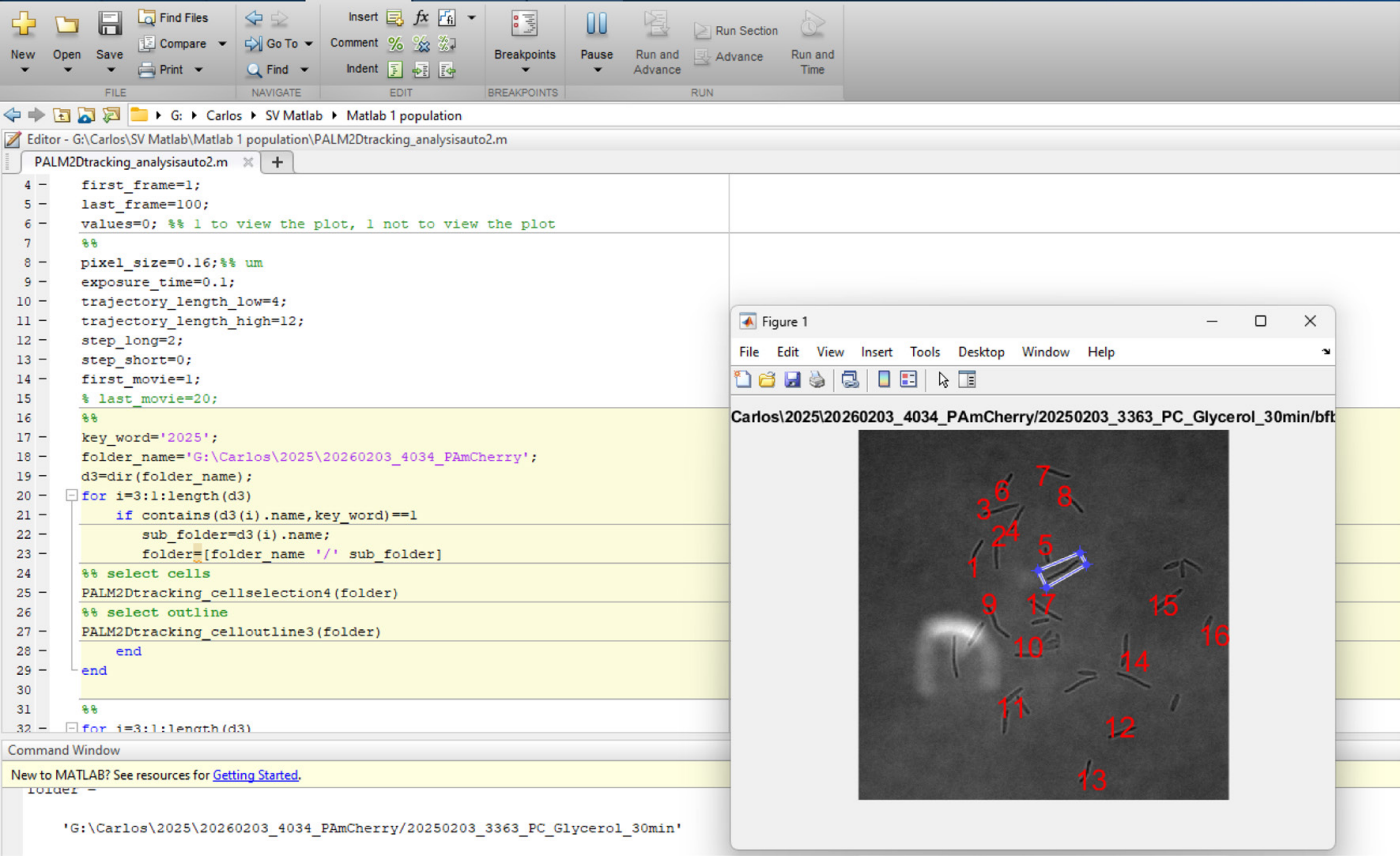
Cell selection. After pressing *Run*, this window appears. Select all cells that contain distinguishable single fluorescent particles by drawing rectangles around them. After cell selection, press *Enter*.

7. In line #18, after folder_name=, copy and paste the name of the directory to be analyzed. Make sure to use single quotation marks (') around the folder_name (Figure 5).

8. Click *Run*. A cell selection window (Figure 6) will appear. To select a cell, draw a rectangle to enclose it: click once to mark the first vertex of the rectangle, and a dot will appear. Drag the cursor to the second vertex and click again. Repeat this process for the third and fourth vertices to close the rectangle (Figure 6). Once the rectangle encloses the cell of interest, double-click to confirm the selection and proceed to the next cell. A red number will appear on top of each selected cell. Repeat this process for each cell individually.

*Notes:*

*1. The Optimization Toolbox feature must be installed in MATLAB to perform this step.*

*2. For the best result, choose the cells that contain distinguishable single fluorescent particles. To reduce human bias, we recommend comparing independent analyses performed by two users. Our previous work shows that different users may choose different cells and count varying numbers of particles; however, these differences typically do not lead to significant changes in the calculated diffusion coefficients.*

**Figure 7.**
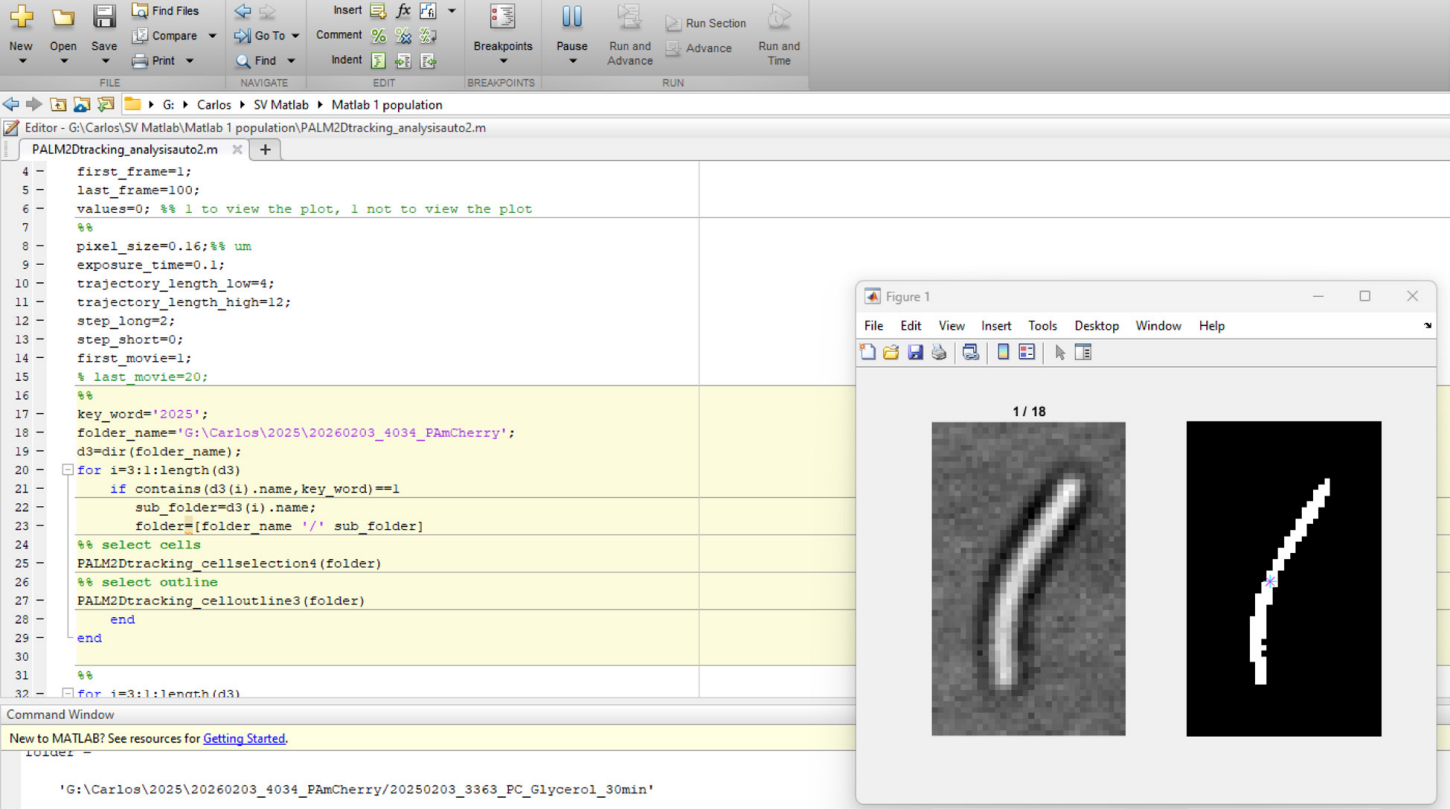
Cell segmentation. After pressing *Enter* in the cell selection window, a new window will appear to outline each selected cell. In the image on the right, click on the white region (the cell) to select it. An asterisk will appear to confirm the selection. Repeat this process for all selected cells.

9. After all cells have been selected, another window will appear (Figure 7), displaying the brightfield image of each selected cell on the left. On the right, the cell surface detected by the program after segmentation (the process of defining cell boundaries) is shown as a white mask. Click the mask (white region) and an asterisk will appear to confirm your selection. Press *Enter* to move to the next cell. Repeat this process for each cell individually.

*Note: This step allows the users to perform quality control on the identification of cell segmentation. Some cells might not be segmented well due to the noisy background and debris nearby. Discard ill-segmented cells by not clicking on them. Press* Enter *to move to the next cell.*

**Figure 8.**
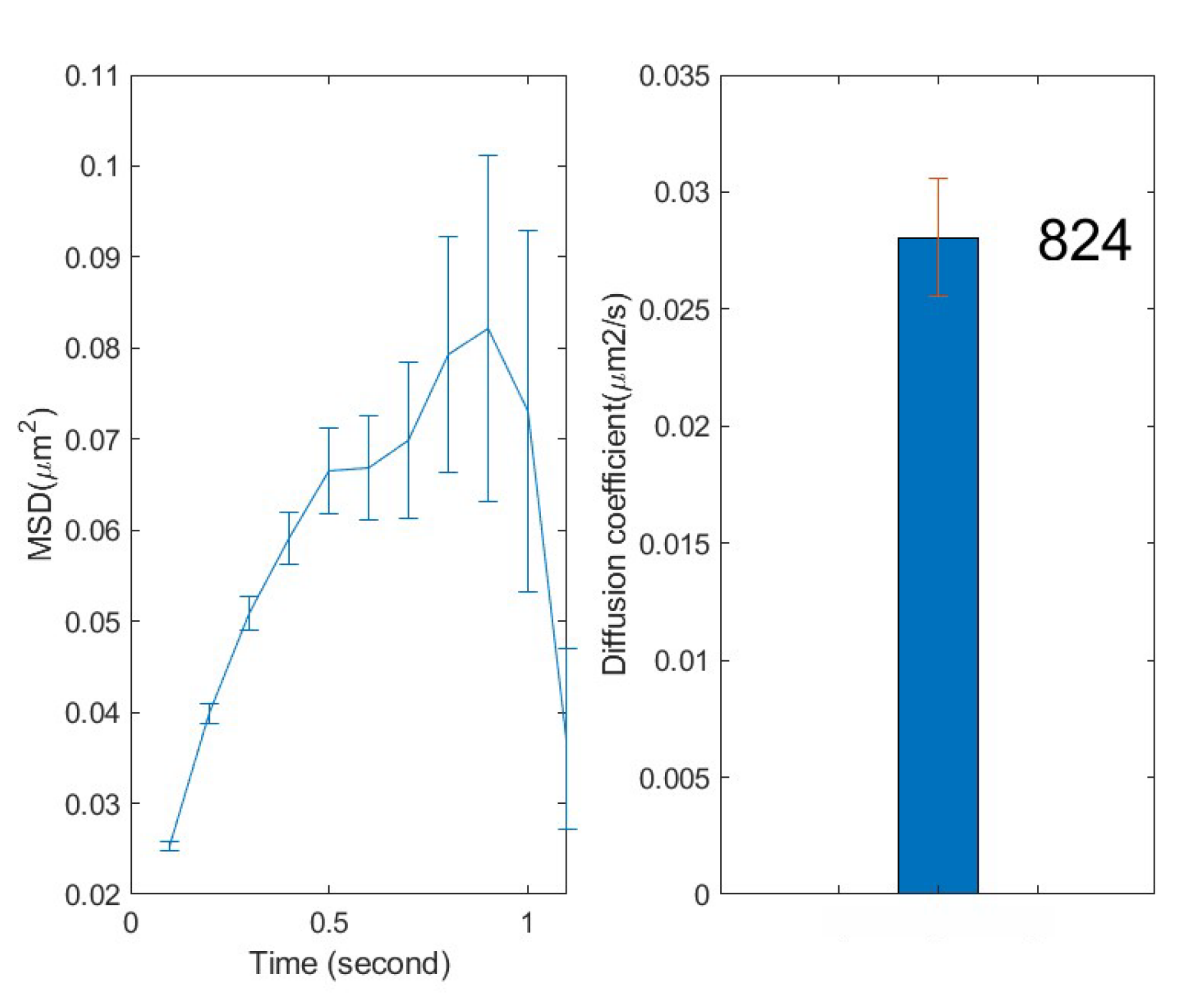
Results of diffusion analysis. The left panel shows the mean square displacement of the particles, while the right panel displays their diffusion coefficients in the cells. The number of particles analyzed is displayed beside the bar. The error bar is calculated from the standard deviation of 1,000 bootstrap samples [1].

10. After all cell outlines have been selected, the program will automatically run. Depending on the computer and data size, it may take a few hours before the analysis completes.

11. Once the analysis completes, a new result window (Figure 8) will appear, which is automatically saved in the data directory as a file named “bf_hist.tifff.” The left panel of this image is a plot of mean squared displacement (MSD) at the population level as a function of time lag. On the right is the diffusion coefficient with the particle number on top and an error bar calculated from the standard deviation of 1,000 bootstrap samples. In our experience, a difference between diffusion coefficients of >0.005 is considered significant [1,4]. The calculated diffusion coefficient, error, and number of particles are saved in a file named “bf_diffusion_coefficient.txt.” All individual coefficient numbers can be found in a different file named “bf_d_individual.txt.”

## Validation of protocol

This protocol has been used and validated in the following research articles:

• Fu et al. [1]. MotAB-like machinery drives the movement of MreB filaments during bacterial gliding motility. *Proc. Natl. Acad. Sci. U.S.A.* (Figure 2B; Figure 3B–D; Figure 4A, C, D).

• Zhang et al. [4]. Coordinated peptidoglycan synthases and hydrolases stabilize the bacterial cell wall. *Nat Commun* (Figure 5A–C, E–F; Figure 6A–C; Figure 7A–C).

• Ramírez et al. [2]. A lytic transglycosylase connects bacterial focal adhesion complexes to the peptidoglycan cell wall. *eLife* (Figure 4B).

• Ramírez et al. [3]. A novel mechanism for bacterial sporulation based on programmed peptidoglycan degradation. *eLife* (Figure 4B, C).

In Zhang et al. [4], western blots were performed to validate the relationship between bound PG enzyme and diffusion coefficient. DacB-PAmCherry in wild-type, untreated vegetative cells has a diffusion coefﬁcient of (2.72 ± 0.37) × 10^-2^ μm^2^/s (n = 1,698 particles), whereas DacB^S75A^, the DacB variant that lacks the serine in its active site, displays reduced motility, with a diffusion coefﬁcient of (2.1 ± 0.49) × 10^-2^ μm^2^/s (n = 1719 particles). To validate the connection between DacB dynamics and its binding to PG, periplasmic domains of DacB and DacB^S75A^ were purified. After a 1-h incubation with puriﬁed *M. xanthus* PG and a brief centrifugation, both proteins precipitated with PG. Compared to the wild-type protein, a signiﬁcantly bigger population of DacB^S75A^ enriched in the pellet, indicating increased binding to PG [4]. Taken together, increased immobile population and decreased diffusion coefﬁcients of DacB particles strongly correlate with their enhanced binding to PG.

## General notes and troubleshooting

In response to different stress factors and genetic backgrounds, several *E. coli* [39] and *M. xanthus* [2–4] strains produce consistent, reproducible shifts in diffusion coefficients, making them useful controls for validating this method.

In *E. coli*, PAmCherry–PBP1A exhibits a diffusion coefficient >2.0 × 10^-2^ μm^2^/s. In contrast, in a strain that lacks LpoA, a PBP1A-specific activator, the diffusion coefficient increases to >5.0 × 10^-2^ μm^2^/s [39]. These strains are well-suited for testing this method.

In the *M. xanthus* strain BN312, DacB–PAmCherry shows a diffusion coefficient of (2.72 ± 0.37) × 10^-2^ μm^2^/s. Following treatment with moenomycin, the value decreases to (1.7 ± 0.40) × 10^-2^ μm^2^/s [4], further supporting antibiotic treatment as a reliable control for detecting diffusion changes.
